# Deep ancestry of collapsing networks of nomadic hunter–gatherers in Borneo

**DOI:** 10.1017/ehs.2022.3

**Published:** 2022-02-21

**Authors:** J. Stephen Lansing, Guy S. Jacobs, Sean S. Downey, Peter K. Norquest, Murray P. Cox, Steven L. Kuhn, John H. Miller, Safarina G. Malik, Herawati Sudoyo, Pradiptajati Kusuma

**Affiliations:** 1 Santa Fe Institute, Santa Fe, California, USA; 2 Complexity Science Hub Vienna, Vienna, Austria; 3 Department of Archaeology, University of Cambridge, Cambridge, UK; 4 Complexity Institute, Nanyang Technological University, Singapore; 5 Department of Anthropology, Ohio State University, Columbus, Ohio, USA; 6 Department of Linguistics, University of Arizona, Tucson, Arizona, USA; 7 School of Natural Sciences, Massey University, Palmerston North, New Zealand; 8 Te Pūnaha Matatini, New Zealand Centre of Research Excellence for Complex Systems, Auckland, New Zealand; 9 School of Anthropology, University of Arizona, Tucson, Arizona, USA; 10 Social and Decision Sciences, Carnegie Mellon University, Pittsburgh, Pennsylvania, USA; 11 Laboratory of Genome Diversity and Diseases, Eijkman Institute for Molecular Biology, Jakarta, Indonesia

**Keywords:** Hunter–gatherers, prosocial behaviour, Borneo, Austronesia, song language

## Abstract

Theories of early cooperation in human society often draw from a small sample of ethnographic studies of surviving populations of hunter–gatherers, most of which are now sedentary. Borneo hunter–gatherers (Punan, Penan) have seldom figured in comparative research because of a decades-old controversy about whether they are the descendants of farmers who adopted a hunting and gathering way of life. In 2018 we began an ethnographic study of a group of still-nomadic hunter–gatherers who call themselves Punan Batu (Cave Punan). Our genetic analysis clearly indicates that they are very unlikely to be the descendants of neighbouring agriculturalists. They also preserve a song language that is unrelated to other languages of Borneo. Dispersed travelling groups of Punan Batu with fluid membership use message sticks to stay in contact, co-operate and share resources as they journey between rock shelters and forest camps. Message sticks were once widespread among nomadic Punan in Borneo, but have largely disappeared in sedentary Punan villages. Thus the small community of Punan Batu offers a rare glimpse of a hunting and gathering way of life that was once widespread in the forests of Borneo, where prosocial behaviour extended beyond the face-to-face community, facilitating successful collective adaptation to the diverse resources of Borneo's forests.

## Graphical abstract

**Figure:**
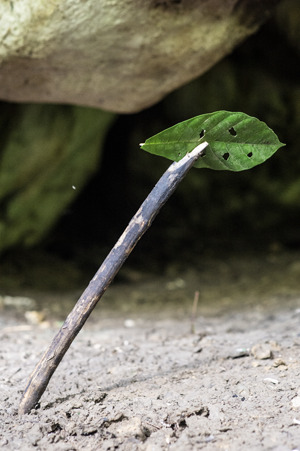

**Social media summary:** Ancient networks of communication among mobile groups of hunter–gatherers in the forests of Borneo are collapsing.

The Punan were the traditional hunter–gatherers of Borneo, but long-term resettlement programmes, deforestation and changing economic conditions have greatly reduced full-time hunting and gathering, making most communities largely sedentary (Guerreiro, 2004; Kaskija, 2002, 2007; Sercombe & Sellato, 2007; Hose & McDougall, 1912). A comprehensive census of the Punan of East Kalimantan in 2003–2004 by researchers from the Center for International Forestry Research counted 8,956 persons. The authors estimated that their census included more than 90% of the total Punan population of East Kalimantan. However, they also noted that ‘the possibility cannot be excluded that because of isolation, some Punan Basap/Batu might still exist in the Berau region’ (Sitorus et al., 2004). Our knowledge of the existence of an isolated community who call themselves Punan Batu (Cave Punan) came about unexpectedly in May 2018 during a medical survey of resettled Punan communities in Northeast Borneo (Figure 1a). A Punan leader from a resettlement community told us that he had recently encountered a community of Punan who live as nomadic hunter–gatherers, and offered to bring us to meet them. In July 2018 he guided us to a rock shelter where a small number of Punan Batu were encamped, and we accepted their invitation to camp nearby. Many Cave Punan spoke Malay as well as their own languages, and from them we learned that they number about 30 families, a few largely sedentary but most living as mobile foragers. In a few days, other Punan Batu joined us. With them we visited other camps and rock shelters, and expanding oil palm plantations that border their forest.

**Figure 1.**
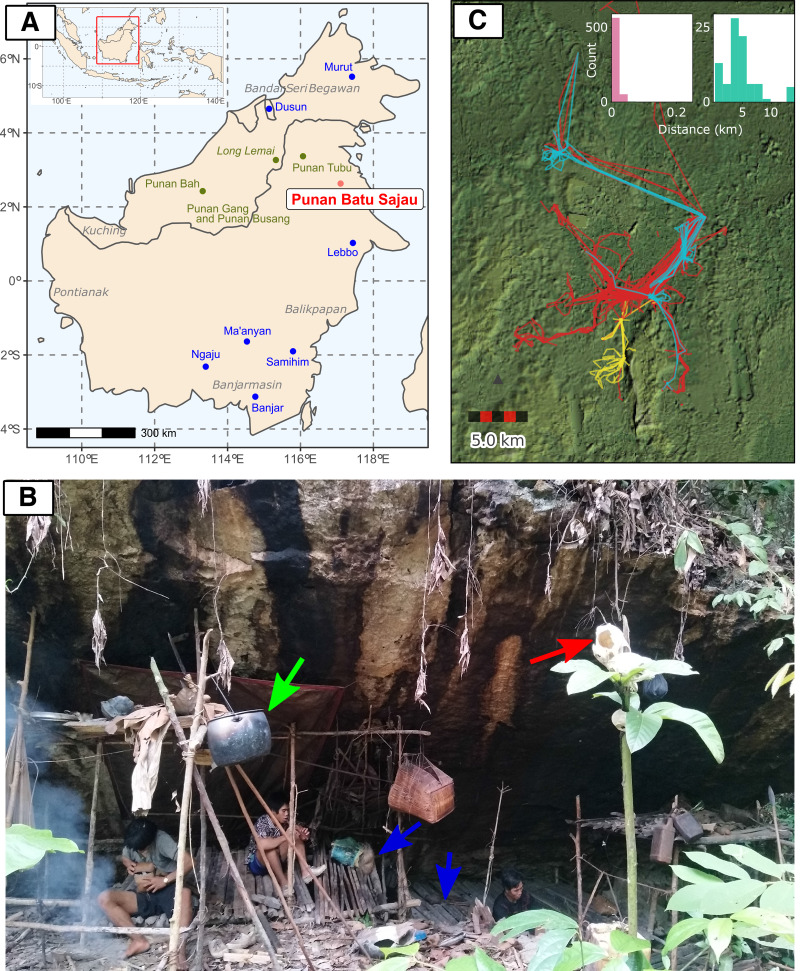
(a) The location map of the Punan Batu community (red) and agricultural groups (blue) included in the genetic analysis, as well as the approximate location of other Punan populations discussed in the text (dark green). (b) A photograph of an active Punan Batu rockshelter and family members of Punan Batu. Blue arrows indicate wooden platforms to sit and lie down on. The green arrow indicates an aluminium cooking pot, obtained through trade. The soot on the rock shelter has accumulated from the frequent use of fire underneath. The red arrow indicates the skull of a boar, hunted and consumed a month before this photo was taken, and traditionally displayed in higher place for ritual purpose. (Photo taken by P. Kusuma; October 2019). (c) Thirty-seven GPS tracks from 27 different Punan adults collected over three 4 week periods, coloured by individual sex (red, male (20); blue, female (three); yellow, not recorded (four)). The inset shows the distribution of distances travelled between campsites over a total of 713 consecutive nights in the second and third periods (also see Figure S1b). People either stayed at the same or at a nearby site (left; move *<* 250 m, *n* = 626) or moved to a different location (right; move 250 m, *n* = 87). When moving camp people moved on average 4.56 km (standard 2.96 km, range 0.32–13.69 km). Long straight lines on the map are produced by canoe journeys. The focal sites in the centre of the map include more established sites and, to the south, important caves and rock shelters.

Many Punan live in remote upland regions of Borneo, but the dense coastal forests of the Cave Punan are located near the town of Tanjung Selor, within the traditional territory of the former Sultanate of Bulungan. In subsequent visits we learned that a descendant of the Sultan (the Datuk) maintains an occasional trading post on the Sajau river where he trades goods for forest products. The Cave Punan we spoke with told us that most of them very seldom leave the forest, and that the availability of forest resources for trade has sharply declined. Some wear clothes fashioned from tree bark, especially for hunting, but most now wear used clothing obtained from the Datuk. These initial observations prompted several questions: do all people who come to trade with the Datuk regard themselves as Cave Punan? Are they mobile foragers? What is their relationship to each other, to the Datuk and to neighbouring Punan and tribal farming communities, both now and in the past? To answer these questions we made repeated visits to Cave Punan forest camps and rock shelters, and gathered additional data on their mobility using GPS and accelerometers. To investigate their ancestry and relationship to one another and neighbouring groups, we combined multiple independent lines of evidence from ethnology, population genetics and linguistic analysis. This approach has recently provided new insights into the origins and ancestry of other societies in the region, revealing for example that the Sama sea nomads of the Sulu archipelago share genetic ancestry with other Philippine groups who do not self-identify as Sama or speak a Sama-related language (Larena et al., 2021). Here they enable us to provide the first comprehensive cultural–genetic–linguistic study of the only known group of Punan hunter–gatherers who remain continuously mobile.

## Results

### Are the Punan Batu mobile foragers?

Over the course of our initial fieldwork, we visited 12 occupation sites, including forest camps (three), riverside shacks built by the Datuk (two) and rock shelters (seven – three occupied and four unoccupied). The Punan Batu travel continuously in small family or multi-family groups between rock shelters, caves and forest campsites (Figure 1b). The composition of these groups is fluid. Caves are not personal property: anyone may stay at any cave or rock shelter. During our discussions with the Punan Batu, they indicated that their lifestyle and identity are strongly distinct from neighbouring non-Punan Dayak groups (who are agriculturalists). This tallied with our observations of their foraging behaviour and movements. We directly observed only hunting and gathering activity. This is consistent with the oft-repeated claim by the Punan Batu with whom we travelled and camped that they are mobile hunter–gatherers, and that hunting and gathering are daily activities. Although wild pigs (*Sus barbatus*) are a focus of hunting, a wide range of other species are also taken, including deer, monkeys, turtles, fish, frogs and birds. The skulls of animals are displayed prominently in and around camps and in crevices in rock shelters. Most hunting relies on dogs and spears; blowguns were traditional but are now rarely used. The Punan Batu also gather a wide variety of forest tubers and fruits. Meat is immediately shared by everyone present at a camp, and plant foods are also shared as needed.

In addition to hunting and gathering, like other indigenous peoples of Borneo, the Punan Batu gather traditional forest products to trade, such as edible birds’ nests. A century ago, the exclusive right of the local Sultanate to edible birds’ nests in the region was recognised by the Dutch colonial administration. The swiftlets nest in caves in the forests of the Punan, but a descendant of the Sultanate, the Datuk, claims the hereditary right to harvest them. The Punan collect the birds’ nests and other forest products (fragrant gaharu wood, wild honey) and bring them to the Datuk's seasonal trading post on the river. The Datuk confirmed that only Cave Punan visit his trading post. He also echoed the Punan's frequent observations that the birds’ nests and gaharu are now nearly gone, and there are fewer honey trees, which they attribute to loss of forest cover caused by the expansion of logging and oil palms, leaving the Punan with little to trade.

We also observed small, apparently experimental, gardens near four of the 12 occupation sites we visited, ranging in size from around 10 × 10 to 15 × 20 m. The gardens were planted with taro, cassava, bananas, pineapple, papaya, sugarcane and some corn. We observed very little tending of the gardens, which appear to comprise a risk mitigation strategy given the increasingly uncertain availability of forest foods. The Punan say that when groups are camped near a garden, if food is available, it is shared with everyone. Interviews revealed interesting cultural taboos: one person volunteered that he had also planted a small garden but was concerned that it would trouble his ancestors, and gave it up. Others mentioned that gardens could bring bad luck. Gardening is small scale and occasional. Unlike with many settled Punan, the growing of rice is very rare – only two families reported recent experiments with rice, and the only rice consumed was obtained from the Datuk, who observed that the Punan Batu only started to eat rice significantly after 1993 (Table S4). While the Datuk's rice is relatively cheap thanks to government subsidies, it is awkward and heavy to carry in the rough terrain of the dense forest.

**Figure 2.**
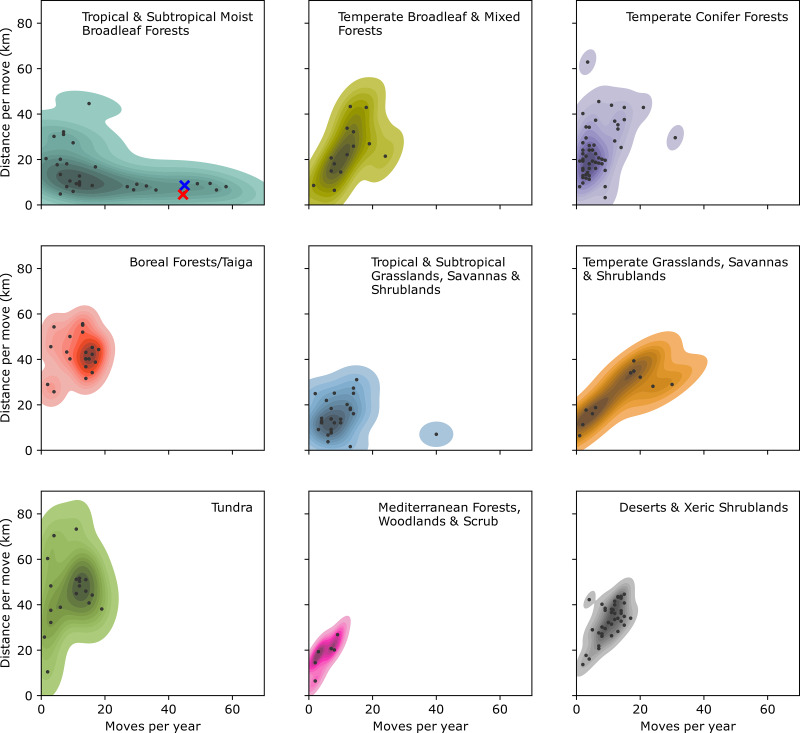
Distribution of movements per year and movement distance for hunter–gatherer societies grouped by ecoregion, as reported in the Binford Hunter Gatherer dataset D-Place (2018a) and Kirby et al. (2016) (see Methods). The Punan Batu (top left, red cross) show a similar high frequency of movements but slightly reduced movement distance compared with mid-nineteenth century data on Western Penan communities (blue cross).

To confirm our observations and quantify the mobility of the Punan Batu, we used portable GPS and accelerometers to map the movements of several dozen individuals in the forest for three periods, each lasting about 4 weeks. Analysis of distances covered over 713 consecutive person-nights indicated movements between camps every 8–9 days, with camps located on average 4.5 km apart (Figure 1c; Figure S3). To examine the mobility patterns of the Punan Batu relative to other broadly defined hunter–gatherer populations, we compared our data with the Binford Hunter Gatherer dataset (see Methods). We visualised how the number of movements per year and movement distance vary among societies according to ecoregion annotation (332 societies, nine ecoregions) (Figure 2). The Punan Batu show extremely high mobility within the variation of their tropical forest ecoregion, with similarly frequent but somewhat shorter (4.56 vs. 8.58 km) movements as reported for the Western Penan society in the Binford dataset based on mid-nineteenth century ethnographies. The Binford data confirms that very frequent, short-range movements comprise a characteristic behaviour of a subset of tropical forest hunter–gatherer societies only. In this context, the Punan Batu appear as continuously mobile foragers who supplement their diet with food obtained by trading forest products with the Datuk. The small gardens we observed provide limited food and are not a major focus of daily activity, strikingly unlike the gardens of neighbouring agriculturalists and sedentary Punan communities.

### Demographic history of the Punan Batu

The mobility networks and our own interviews indicate that the Punan Batu have limited contact with neighbouring farmers, the descendants of Austronesian-speaking Neolithic peoples who began to arrive in Borneo about 4000 years ago (Xu et al., 2012; Bellwood, 2014) and who retain a substantial mainland Southeast Asian genetic signature (Mörseburg et al., 2016; Lipson et al., 2014). However, sedentary resettled Punan communities often live in close association with agricultural populations, and their genetic relationships are unknown. To determine whether the Punan Batu might be recent descendants of these agricultural groups, and to assess the extent of their genetic isolation in recent times, we obtained high-density genotype data for Punan Batu individuals and compared them with publicly available genetic data representing a range of indigenous groups from Borneo and the wider region (Table S1).

**Figure 3.**
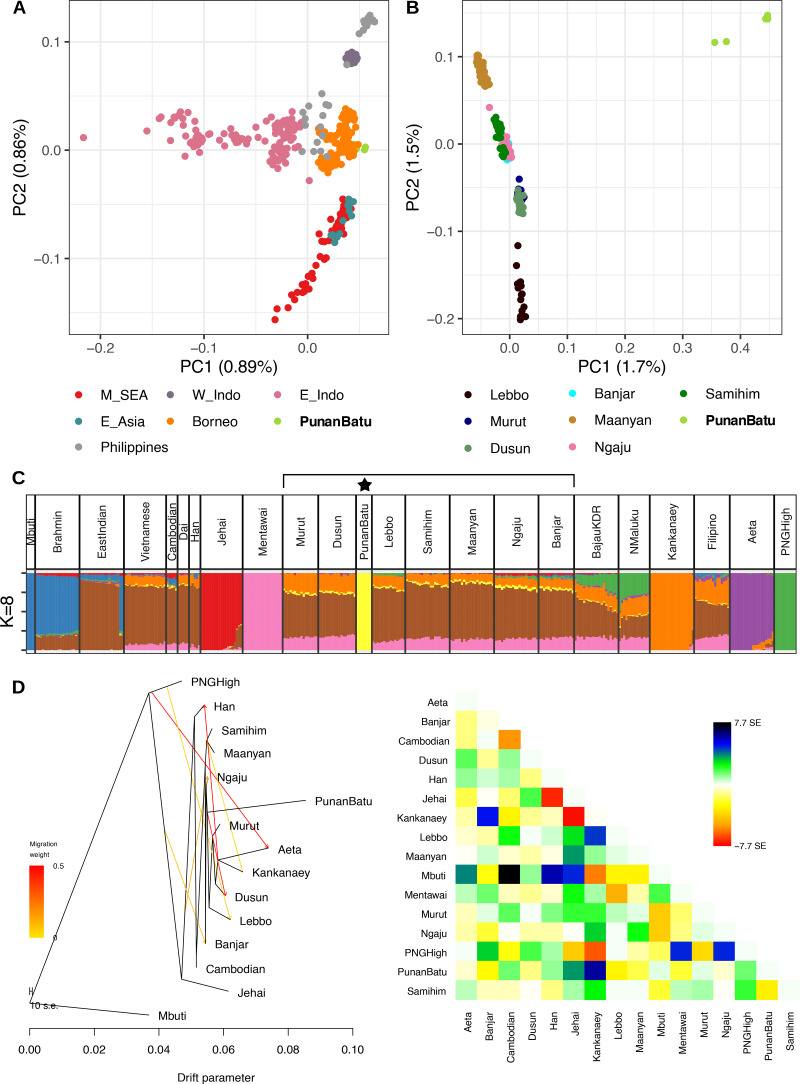
(a) Principal component analysis including East Asian, Mainland Southeast Asian, and Island Southeast Asian populations, and (b) populations within Borneo. (c) ADMIXTURE analysis of regional populations showing the unique Punan Batu ancestral component at optimal *K* = 8 components. (d) TreeMix analysis on seven migration nodes (99.4% variance explained) and its residual plot.

Our analyses show that the Punan Batu form a genetic cluster distinct from neighbouring tribal peoples of Borneo (Figure 3). The Punan Batu are well differentiated from all other indigenous groups, including neighbouring agriculturalists (Figure 3b). Indeed, in a principal component analysis, the Punan Batu are genetic outliers on both the first (south interior Bornean agriculturalist Ma'anyan–north Bornean agriculturalists–Punan Batu) and second axes (east Bornean Lebbo’ gatherer–horticulturalists–north Bornean agriculturalists–Punan Batu and Ma'anyan). Estimating the genetic ancestry of individual people using ADMIXTURE (Alexander et al., 2009) also reveals a single unique Punan Batu-specific ancestral component (Figure 3c). This component emerges from the composite ancestry characteristic of Borneo (Lipson et al., 2014), and is linked at a deeper level to mainland Southeast Asian and East Asian groups who speak very different languages (e.g. Dai, Han, Vietnamese; Figure S2).

To interrogate the relationship between the Punan Batu and other regional groups we ran a TreeMix analysis (Pickrell & Pritchard, 2012). This constructs a bifurcating phylogenetic tree based on patterns of population divergence, before refining it using migration nodes to increase the variance explained and eliminate residual (unexplained) differences between population pairs. While all trees were effective at describing genetic diversity (*>*97.5% variance explained), significant residuals remained even for trees with many migration nodes (*m* = 7). We therefore present both this tree (Figure 3d), and models with both more and fewer migration nodes for comparison in the Supplementary Information (Figure S6). Bornean diversity is nested within mainland Southeast Asian diversity, and is an outgroup to classic modern proxies of Austronesian expansion-related ancestry (Kankanaey/Mentawai). The Punan Batu sit deep within Bornean diversity; interestingly, and in contrast to all other sampled Bornean groups, they do not have any migration nodes from either mainland Southeast Asia or the Kankanaey branch (i.e. known descendants of agricultural populations). Our analysis supports previous reports of significant complexity in Bornean genetic ancestry, but highlights that the Punan Batu are isolated within this.

**Figure 4.**
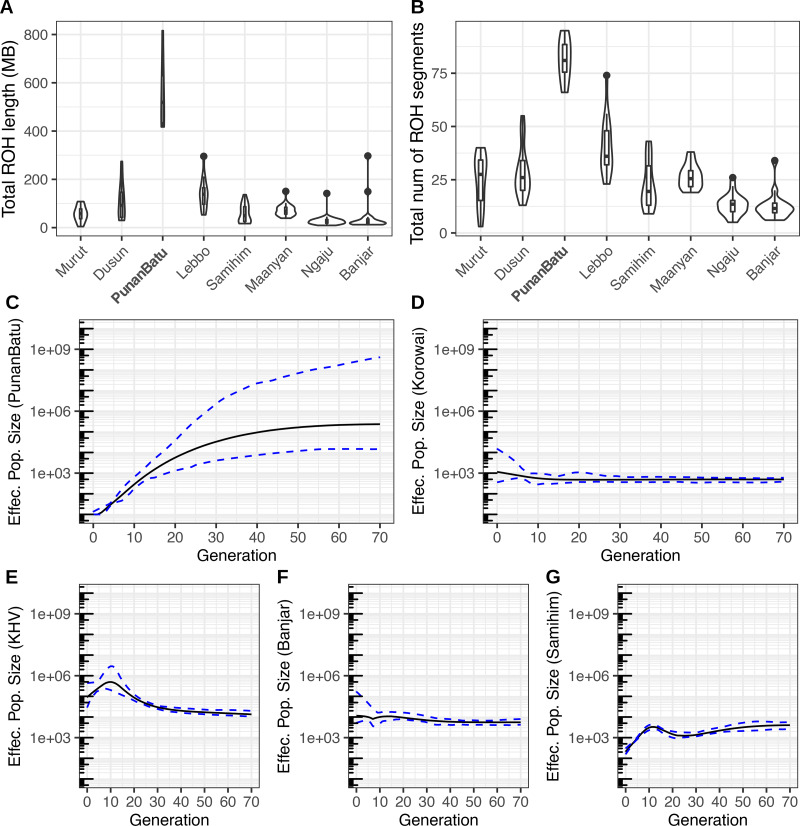
(a. and b) Total runs of homozygosity length (left) and number of segments (right) in Punan Batu individuals compared with other Borneo populations. (c–g) Effective population size dynamics over the last 70 generations in Punan Batu and Korowai hunter–gatherers, and Kinh, Banjar and Samihim agricultural populations respectively, as inferred using IBDNe.

To further confirm this pattern and its implications for their demographic history, we measured identity-by-descent (IBD), which is a time-scaled measure of recent shared ancestry within the Punan Batu as well as a marker of genetic contacts with outside groups. The Punan Batu show long intra-population IBD segments (Figure 3) and long runs of homozygosity (ROH), both consistent with isolation (Figure 4a,b). The high ROH observed in the Punan Batu mirrors but exceeds a broader pattern of increased ROH in traditional southeast Asian hunter–gatherer populations (Figure S4), suggesting that any impact of camp fluidity or composition on inbreeding avoidance (Hill et al., 2011) is limited by long-term endogamy and low population size. Direct inference of population size dynamics using IBDNe on our high-density genotyping data confirmed our inference, revealing at least 20–30 generations of reduced population size (Figure 4c), in contrast to other hunter–gatherer groups in West Papua (the Korowai) (Figure 4d) and other agriculturalist groups in Vietnam (KHV) and Borneo (Banjar and Samihim) (Figure 4e–h). Inference uncertainty at deeper times mean that we are unable to confidently infer the date of this population decline, and hence whether it was associated with the spread of Austronesian-language speaking groups into Borneo – an interesting but open question. The relative genetic isolation of the Punan Batu is further evidenced by their limited IBD with other groups, with a maximum sharing of 15.6 cM in the Punan Batu–Lebbo’ pair, in striking contrast to the extensive interactions observed for all other non-Punan Borneo populations (each with a maximum pairwise IBD of 23.2–43.2 cM; Figure S5).

The unique ancestral genetic signature in the Punan Batu, and their divergent status over multiple independent genetic analyses, indicates that the Punan Batu have long been genetically separate from nearby agriculturalists and have experienced substantial genetic drift probably owing to a combination of cultural endogamy and long-term genetic isolation. These patterns are not consistent with the hypothesis that the Punan Batu are a recent offshoot of neighbouring agriculturalists who reverted from a farming subsistence strategy. Instead they are strong evidence that these hunter–gatherers have their own distinct demographic history.

### Languages of the Cave Punan

**Figure 5.**
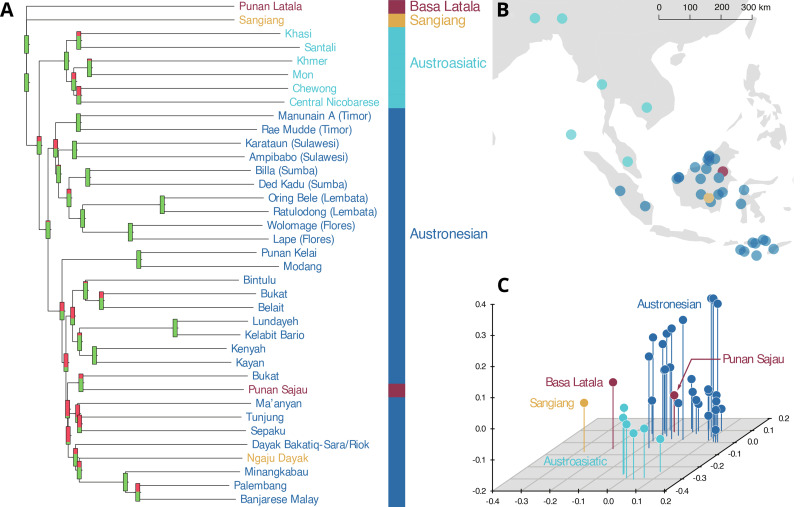
(a) Language tree representing the relationships between Basa Latala, Sangiang, and the Punan and Ngaju Dayak daily languages, in the context of a sample of Austronesian and Austroasiatic languages. The significance of each possible bifurcation is indicated by thermometers representing the proportion of agreement among 1000 bootstrap trees, where all green represents 100%. (b) Location map of populations whose language used in this study. (c) Classical multidimensional scaling (MDS) plot showing the relationship between languages in the sample. Basa Latala and Punan Sajau are drawn in the same colour to indicate that they are both spoken by the Punan Batu. Data available at https://figshare.com/s/e6107567f0ef4a42bf27.

The evidence from language is more complex, but consistent with inferences from the genetic data. The everyday spoken language of the Punan Batu is *Punan Sajau*, a Central Sarawakan Austronesian language which is related to those of their close neighbours, both Punan and Dayak (Smith, 2017b). An internal subgrouping of Punan dialects recognises a primary split between Punan Sajau, Tubu and Bah (the closest Punan neighbours of the Punan Batu, Figure 1) and the other Punan groups (Smith, 2017a). Many Punan also have some knowledge of Malay, the Austronesian trade language common to all Borneo peoples. However, some are also fluent in a hitherto undocumented language which is not spoken, but rather sung, customarily at night in the rock shelters or caves. This song language, *Latala* or *Menirak* (‘to sing’ in the daily Punan language), is known only to the Punan Batu, although it shares some formal features with other ritual song languages of Borneo such as *Sangiang* (Baier & Scharer, 1987), the language of the *first ancestors* employed by the Ngaju Dayaks whose territory lies to the south of the Punan Batu in Central Kalimantan. Figure 5a shows the phylogenetic relationships between these languages, estimated using an optimised linguistic distance (Downey et al., 2017). Besides the two song languages just mentioned, we included other Bornean languages of the Austronesian phylum as well as a sample of Austroasiatic languages (Figure 5b), because the latter are geographically proximate within Southeast Asia, and structural and lexical similarities have been previously noted between Austroasiatic languages and various language groups of Borneo (Adelaar, 1995; Blench, 2010). The language data used in this analysis are available on Figshare (https://doi.org/10.6084/m9.figshare.12466001).

This dual pattern of a daily language, often originally adopted during periods of substantive change in trade relationships, and an unrelated ritual language is also observed in other regions with high language diversity (Crevels & Muysken, 2020). As noted above, the Punan Batu carry genomic components that are localised to Borneo and appear to be old rather than arising recently through genetic drift (Figure 3). The Latala song language, a language isolate, may trace back to the ancestral community that contributed one or more of these Bornean genomic components. Latala is equally remote from both the Austronesian and Austroasiatic language families (Figure 5c; Figure S7), and the only words of Austronesian origin detected so far appear to be the result of intraspeaker transfer from the daily language of Punan Sajau into Latala. Latala is similarly remote from Sangiang (Table S3), which itself is isolated from Austronesian and Austroasiatic languages and displays minimal sharing with *Ngaju Dayak*. Other ritual Bornean song languages are typically closely related to their spoken language, and do not resemble the song language of the Punan Batu. Therefore, Latala appears to be only distantly related to both Austroasiatic, Austronesian and other song languages, as well as everyday Punan Sajau. Latala was not understood in any of the other Punan groups we visited. Unlike Sangiang, it is not used for formal religious rituals. Instead we observed that for the Punan Batu, Latala functions as a *kunstsprache*, a language that was once spoken but is now used exclusively for poetic expression, similar to Homeric Greek, Latin or Old Javanese. The retention of Latala by the Punan Batu is most parsimoniously explained as an ancient cultural inheritance, further evidence for their distinct demographic history.

### Message sticks connect nomadic groups

Our observations and GPS data show that the Punan Batu travel continuously in very small groups, whose composition frequently changes. Yet both the genetic and the linguistic evidence described above indicates that they are not simply a congeries of family units, but a cohesive endogamous society with a shared genetic and linguistic heritage. How are these connections sustained? While travelling with the Punan and mapping their movements in the forest (Figure 1c; Figure S1), we observed the active use of a system of communication that was formerly widespread among nomadic Punan and Penan groups across Borneo, but is rapidly vanishing among sedentary groups: message sticks.

**Figure 6.**
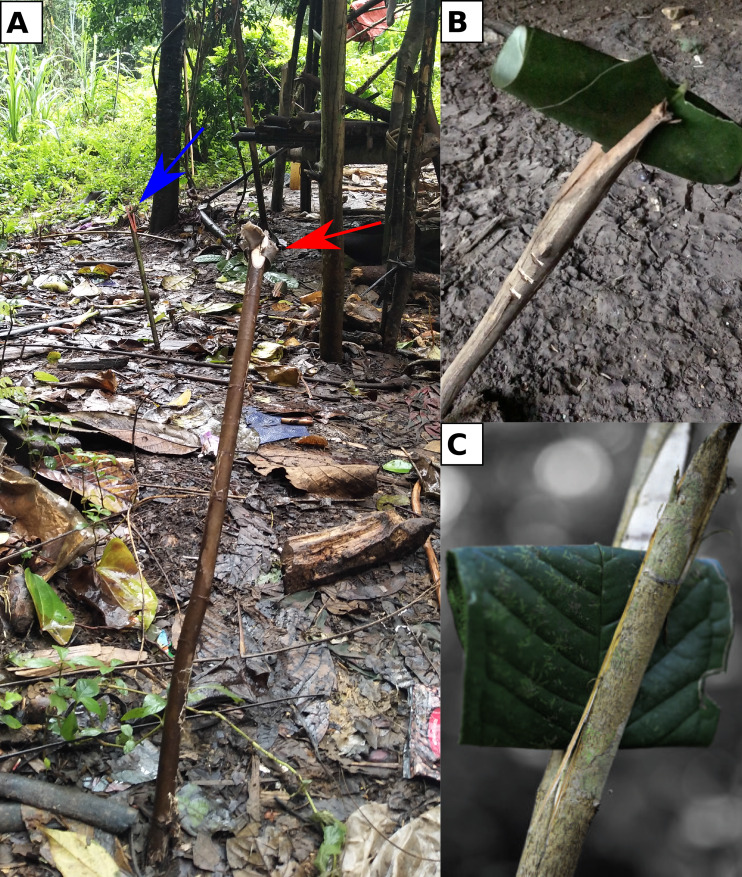
(a) Two message sticks were left near an uninhabited camp in the forest, indicating the direction in which the two different persons/families went. Both sticks carry an object inserted into the notch on top of it, which represents a sign of a person's name (red arrow, a piece of cloth; blue arrow, a small piece of carved wood). The length of the stick indicates the distance. (Photo credit: P. Kusuma, Eijkman Institute.) (b) A Punan Batu rolled leaf signifying ‘I/we are hungry’, a request for aid. (Photo credit: J. S. Lansing, Santa Fe Institute.) (c) ‘I am hungry’, from resettled Penan communities of Long Lemai/Kerong, Upper Baram region of Sarawak. (Photo credit: T. Zaman, University College of Technology Sarawak.)

The Punan Batu are quick to emphasise the difficulties of finding and sharing food, and the importance of knowing the whereabouts of other groups. Message sticks are key to the realisation of both of these goals. When a group decides it is time to move, the members customarily place a stick in the ground whose length and direction indicate where they plan to go. Every adult has a symbol for their name that can be used to identify them on a message stick. The sticks are also used to transmit more complex signals. For example, a rolled-up leaf placed in a cleft signifies an urgent request to bring food to the sender's group (Figure 6). There is also a stick symbol for the presence of disease, used to warn others of the risk of contagion. More symbols point to fruiting trees, and function as invitations. As previously noted, our GPS data show that Cave Punan move camp or spend periods away from their camps every 8 or 9 days, either when local resources have dwindled or on extended visiting or foraging trips. Message sticks provide a way to share vital information for planning these moves, including requests for help, directions to foraging opportunities and warnings of danger, such as disease, to all who can read the signs (the Punan assert that they are neither noticed nor understood by non-Punan). In this way, ongoing communication by message sticks defines the Cave Punan community, sustaining a social network in which each adult is a node.

There is abundant ethnohistorical evidence that the use of message sticks was once widespread among nomadic hunter–gatherers in Borneo. Tribal farming communities in Borneo do not use message sticks, although different systems of signs (viz. *etuu*) are used by some tribal farmers to assert ownership and boundary claims (Janowski & Langub, 2011). However, a 1955 expedition to study the Punan living at the headwaters of the Rejang river in Sarawak, approximately 450 km distant from the Punan Batu, reported that message sticks were in frequent use among mobile groups. Drawings included the rolled up leaf signal signifying a request for food, identical to the message stick used today by the Punan Batu (Arnold, 1958). Arnold (1958) and Harrison (1965) describe two systems of message sticks used by the Punan Busang and Punan Gang in Sarawak. Thus the use of message sticks by nomadic Punan and Penan, attested in many studies beginning in the 1950s, enabled individuals and groups to share information for planning their mobile foraging strategies and social interactions. Among contemporary Punan Batu, a sociolinguistic community comprised of small groups of mobile foragers, the message sticks provide a shared repertoire of signs that is in constant use.

Apart from the Punan Batu, the knowledge and use of message sticks now appears to be rapidly declining in resettled Punan and Penan communities. An example is the Punan Tubu, whose spoken language is most similar to the spoken language of the Punan Batu, although they do not understand the Latala song language. They are a group of about 800 people who lived a semi-nomadic lifestyle until the last 20 years, when they settled down in five small, scattered hamlets in response to incentives from the government, and began to grow upland rice, which has become their dietary staple (Tables S4 and S5; Sercombe & Sellato, 2007; Dounias et al., 2007; Reyes-Garcia et al., 2019). A 2014 study of the mobility of 54 adults in two Punan Tubu villages tracked 116 trips into their gardens and surrounding forests (Maximilien & Napitupulu, 2017). The frequency of these trips is roughly comparable with those we recorded among the Punan Batu, with the significant difference that the Punan Tubu used their villages as more permanent bases, with less need for message sticks. In 2020 we found only one elderly hunter who was regarded by others in his community as competent in their construction and use.

The experience of the Punan Tubu, for whom fluid social networks linked by message sticks have been replaced by sedentary villages, is widely repeated across Borneo. The disappearance of the message sticks signals not only the transition to a sedentary or semi-sedentary lifestyle, but the loss of the networked connections between mobile groups that the message sticks helped to perpetuate, and the social practices they facilitate. To our knowledge, message sticks are no longer in active use except among the Punan Batu. For example, a 2016 survey of the rapidly declining knowledge of message stick signs (*oroo’*) among 80 Penan in the resettlement community of Long Lemai (Sarawak, Figure 1), which was created in the 1950s, found that individuals above the age of 50 years knew more than 40 Oroo’ signs, those between 20 and 30 knew eight signs, and individuals under age of 20 knew just four signs (Tariq et al., 2017).

### Message sticks and prosocial behaviour

A major theme in the anthropology of hunter–gatherers and the evolution of human behaviour is the comparative analysis of cooperation. Food sharing is crucial to hunter–gatherers who rely on high-quality but unpredictable and hard-to-acquire resources (Kaplan & Gurven, 2005). So far, most studies of contemporary hunter–gatherers focus on sharing by individuals in face-to-face interactions, which compare their proclivity to share, contribute or free-ride (Lewis et al., 2014). The message sticks used by the still-mobile Punan Batu reveal a more extensive mode of cooperation that can be clarified by contrasting their practices with those of resettled Punan and Penan living in sedentary and semi-sedentary communities.

For the nomadic Punan Batu, message sticks have diverse functions, whose adaptive significance can be explored through the lens of evolutionary games. Depending on the scenario, various games such as simple coordination and the Prisoner's dilemmas can arise. Messages surrounding disease avoid the spread of disease and illness, allowing individuals and groups to maintain higher levels of fitness overall. We were warned away from one forest camp by such a message. Messages about the direction a group is moving in allow groups to re-establish direct contact or perhaps avoid the over-crowding of limited resources. Messages also focus on the sharing of food. In this ecosystem, food sources, such as fruiting trees, produce non-storable food in abundance for short periods at unpredictable times. In such a case, telling others about the potential harvest does not diminish a group's own access to food while also encouraging the reciprocity of other groups, thus mitigating some of the long term risk in acquiring food. The sequential and repeated nature of the various interactions mediated by the messages also encourages cooperation by enhancing coordination and lengthening the shadow of the future (Axelrod & Hamilton, 1981). Furthermore, the signing of the message sticks makes reputation important, which can encourage cooperation and lower incentives to send false signals. Of course, it is one thing to leave a signal, while it is another for someone, even if they see it, to respond.

Thus the message sticks of the Cave Punan can be viewed as an institution that facilitates both social and social–ecological interactions within their society. This role can be highlighted by comparison with the message sticks of Indigenous Australians, which were used for occasional communications between groups: ‘Carried by special messengers over long distances, their motifs were intended to complement a verbally produced communication such as an invitation, a declaration of war, or news of a death’ (Kelly, 2020).

A more immediate clarification of the functions of message sticks is observable in resettled Punan communities, where they are no longer in frequent use. Numerous researchers have noted that the sharing of food remains widespread among Punan who live in permanent villages, and typically takes the form of demand sharing in face-to-face interactions. People keep track of what is given, and free riding is subject to sanctioning. For example, among the semi-sedentary Punan Tubu, a 2007 study showed that demand sharing is normative, but individuals frequently try to evade it by hiding food and other goods (Cesard, 2007). In contrast, for the nomadic Punan Batu keeping close score of the exchange of goods is impractical. Consumption is a public event, taking place on temporary wooden platforms in rock shelters or in forest camps. We observed no instances of overt demand sharing: food is immediately shared. This is consistent with simulation models predicting that high mobility and fluid membership in small groups can sustain non-producers (free riders, pre-adults and post-productive adults) in relatively high numbers (Lewis et al., 2014). While all societies of foragers encourage food sharing within the group, routinely broadcasting messages to other groups to request assistance, share resources or avoid contagion has not previously been observed elsewhere. In conjunction with the absence of territorial claims and the fluidity of movements between caves and rock shelters, this moral and practical obligation to share institutionalises norms of cooperation that extend between as well as within groups.

## Conclusion: the antiquity of the Cave Punan

The likelihood that the communication networks sustained by the message sticks had vital adaptive significance, in light of the constant mobility of groups of Punan Batu, is strengthened by the evidence for their antiquity. For decades anthropologists have debated whether the Punan are indigenous agriculturalists who experienced recent (Hoffman, 1986) or more ancient subsistence shifts from horticulture to forest nomadism (Sather, 1995; Bellwood, 2017) or instead have greater continuity with pre-Austronesian hunter–gatherers (Sellato, 1994). Our genetic analysis is strongly consistent with only the latter scenario for the Punan Batu, and their retention of the Latala song language is further evidence in support of cultural as well as demographic continuity. The continuing reliance on message sticks that we observed among the Punan Batu is also consistent with the uses described in earlier ethnographies. The genetic evidence further suggests that, in the past, various Punan groups were connected by networks of message sticks, implying a role for between-group prosocial behaviour similar to that still practised by the Cave Punan.

Recently archaeologists and palaeoecologists have begun to question the simple two-stage model of pre-Austronesian foraging followed by Austronesian farming in Borneo, known as the ‘staircase model’. Interdisciplinary investigations of the archaeology and palaeoecology of the Niah Caves reveal a 50,000 year record of human interactions with the rainforests on the coastal plain of Sarawak, which include patterns of interactions that do not have simple analogies with the subsistence activities of present-day rainforest foragers and farmers (Barker et al., 2017). The existence of a dichotomy between hunter–gatherers on one hand and agriculturalists on the other has been characterised as unhelpful for understanding patterns of activity that are far too complex, nuanced and locally contingent to be attributed neatly to simple economic categories or narratives (Hunt & Rabett, 2014). In this context, the evidence presented here for the antiquity of the Punan Batu, their song language and message sticks is not easily explained from the standpoint of cultural reversion. Their genetic, linguistic and cultural data all contrast with known cases of hunter–gatherers who originated from an agricultural group and then adopted a hunting–gathering mode of subsistence such as the Mlabri of northern Thailand (Oota et al., 2005; but see Waters, 2005 for an alternative hypothesis on the origins of the Mlabri). Other hypothesised examples of cultural reversion, such as the Guaja’ and other lowland Amazonian groups (Balee, 1999), and the Sirionó of Bolivia (Stearman, 1984), are controversial, as it is not clear whether these groups are descended directly from earlier hunter–gatherer groups or whether they indeed have undergone cultural reversion. Detailed genetic analyses, as carried out here for the Punan Batu, may shed further light on these cases.

In recent years (roughly two generations) the Cave Punan have begun to try to supplement their diet with gardens and foods obtained by trade with the Datuk. While the GPS data show that the Punan Batu continue to live as mobile foragers, their ability to continue this way of life is imperiled by the expansion of both oil palm plantations and commercial logging on the borders of their homeland, which they associate with declines in honey trees, swiftlet nests and the availability of game animals (Figure S1). The mobility data show that reliance on hunting and gathering for subsistence requires a relatively large range, and small dispersed groups which continuously share information, as the Punan Batu are acutely aware. Several Punan composed and sang songs to us in Latala evoking the loss of their forests and asking for help.

## Methods

### Ethical approval

Biological sampling was conducted by the Eijkman Institute for Molecular Biology (EIMB), with the assistance of Indonesian Public Health clinic staff, following protocols for the protection of human subjects established by the EIMB. All samples were collected with informed consent. Collection, use of samples, mobility study (GPS and accelerometer employment) and biological data analysis were approved by the Eijkman Institute Research Ethics Commission, Indonesia (EIREC no. 122, 2018). Ethnographic observation, filming, social and linguistic surveys and the use of GPS and accelerometers were approved by the Institutional Review Board of the University of New Mexico (IRBNet ID no. 1290975-4).

### Biological samples

Volunteers were surveyed for language affiliation, current residence or location of camps/rock shelters for the Punan Batu, familial birthplaces and a short genealogy of three generations to ensure regional and ethnolinguistic ancestry. A total of 11 Punan Batu samples with no known non-Punan Batu ancestors in the parent, grandparent or great grandparent generations were analysed from the Punan Batu community in Bulungan Regency, North Kalimantan Province (Figure 1; Table S1). We also added DNA samples from Mentawai, West Sumatra (*n =* 18) and Mappi, West Papua (*n =* 5) as comparative populations. All samples were blood samples, and DNA was extracted using Gentra Puregene Blood Kit (Qiagen, USA) following the manufacturer's protocol. All laboratory works were conducted at the EIMB, Jakarta, Indonesia.

### Genotyping and dataset integration

The samples were genotyped using Illumina Omni 2.5 array, resulting in 2.4 million variants typed across the genome, of which 2.3 million are autosomal variants. A comparative dataset was built from 26 worldwide populations comprising an additional 406 individuals (Table S1). Data quality controls were performed using PLINK v1.9 (Chang et al., 2015): (a) to avoid close relatives, relatedness was measured between all pairs of individuals within each population using an IBD estimation (upper threshold 0.25 i.e. second-degree relatives) and confirmed using KING-robust (upper threshold 0.084; Manichaikul et al., 2010) in the SNPRelate R package (Zheng et al., 2012), leading us to remove three Punan Batu individuals with a final Punan Batu dataset of six males and two females; (b) Single Nucleotide Polymorphism (SNPs) that failed the Hardy–Weinberg exact test (*p* *<* 10*^−^*^5^) in each population were excluded; (c) samples with an overall call rate *<*0.99 and individual SNPs with missing rates *<*0.05 across all samples in each population were excluded. Genotypes were phased with EAGLE v2.4.1 (Loh et al., 2016) using the Indonesian Genome Diversity Panel pilot phased data (Jacobs et al., 2019) as a reference panel, which covers more variants in island Southeast Asia and, importantly, Indonesian populations. For certain analyses, we applied linkage disequilibrium SNP pruning using PLINK v1.9 (Chang et al., 2015). Pruning was performed in 50 SNP sliding windows with a step size of five SNPs, and SNPs with *r*^2^ *>* 0.2 were removed. The final dataset (before pruning) contains 227,102 SNPs.

### Genetic analyses

Population structure was evaluated using a suite of different programs/algorithms to make robust inferences. Principal component analyses were built using the SNPRelate R package (Zheng et al., 2012) after linkage disequilibrium SNP pruning, applying a stricter PI HAT threshold (PI HAT *<* 0.1) and without removing any outliers (Figure 3). ADMIXTURE v1.3 (Alexander et al., 2009) was also used to observe the population structure by classifying individuals into *K* clusters based on genetic similarity using maximised likelihood with high-dimensional optimisation. Ten randomly seeded runs were performed for each number of ancestral populations (*K* = 2–15), and the results within each *K* were summarised with CLUMPP v1.1.2 (Jakobsson & Rosenberg, 2007). Cluster *K* = 8 was shown to have the lowest cross-validation error value, and was therefore chosen as the best model describing the dataset. To obtain a broad view on the genetic diversity of the Punan Batu, we performed intra- and inter-population pairwise IBD analysis from haplotype data. Haplotype sharing using the Refined IBD algorithm of Beagle v4 (Browning & Browning, 2013) was computed to estimate the total number of shared genetic fragments (logarithm of odds ratio *>* 3) between each pair of individuals. The IBD tracts resulting from RefinedIBD were also used to infer the recent dynamics of the effective population size using IBDNe (Browning & Browning, 2015). To make ancestral inferences, we used TreeMix v1.3 (Pickrell & Pritchard, 2012) analysis. We set African Mbuti as the root, and used blocks of 100 SNPs to account for linkage disequilibrium, then allowed migration edges to be added sequentially.

### Mobility analysis

Initial interviews revealed the Punan Batu to be highly mobile, inhabiting flexible camps consisting of single-family or multi-family units that frequently move around the forest according to subsistence and forest product resource availability. Movements may occur between camps, or entire families may move to a new campsite or rock shelter. To better understand mobility, we asked a sample of 27 different Punan adults to wear GPS units (MobileAction i-gotU GT-600) for a 1 month period following three field visits, in October 2018 (five individuals) and March 2019 (15 individuals; two units were distributed to each individual in order to profile GPS errors; and finally in June 2019 (17 individuals). Most individuals (20) were male, with some women (three) and a small number (four) for whom the sex was not recorded as the units were distributed to a family without exact knowledge of which unit went to which individual. Individuals were identified through a contact network, such that they are not a random sample of the population. The data can nevertheless be highly informative about mobility behaviour and minimal population range.

The GPS units were programmed to start recording the location after the field team left the area, and to attempt to retrieve a GPS reading every 30 minutes. We asked individuals to return the units through the contact network at their own convenience after the 1 month period. Individuals were asked not to swap GPS units, and to wear them as often as possible and especially on trips out of the camps. This sampling strategy avoided undue impact on mobility from interactions with the field team or the device return process. The battery life of the GPS units was sufficient to record several weeks of data (e.g. average 24.0 days in the March 2019 collection). During the first collection, in which GPS units were worn on a wristband, several GPS units were lost or broken owing to the rainforest environment. In subsequent collections we attached the GPS units to a canvas belt, which lead to a low failure rate despite the difficult conditions (*<*5%).

We first used the data from the second collection, in which two GPS units were distributed to each individual, to assess the accuracy of the devices in field conditions. We compared the readings of the two devices worn by each individual whenever they reported a GPS reading within 1 minute of each other (*n* = 438 records). The median difference between near-simultaneous GPS readings was 27.7 m (Figure S1a), with a small number of outlier readings (11 of 428 readings had a difference *>*200 m). These between-unit error results confirm that the GPS units are highly accurate despite the forested environment, with occasional erroneous readings. We note that the GPS units were fixed to belts securely and that all units were returned on the same belts that they were distributed on, such that we believe these infrequent errors to be a property of the GPS devices rather than redistribution of GPS units between individuals.

Using one GPS track from each individual (selecting the track with more points for individuals with two tracks), we prepared the full dataset by trimming the start and end of data collections to avoid periods influenced by researcher activity/the return of units, and masking points that were (a) over 6400 s apart (434 points masked), then (b) over 15 km apart (six points masked, owing to three single-point long-range GPS errors). We visualised tracks between unmasked points (Figure 1c and Figure S1c–e).

We sought to assess the frequency of local movement between camps by retrieving GPS data from consecutive nights for each individual, using the data from March and June 2019. Nights were defined as between 10 p.m. and 6.30 a.m., a period of extremely limited movement activity. For each individual, we averaged the location of GPS points for each night and calculated the difference between this centroid location and the centroid location for the following night. Among all individuals we were able to study 713 pairs of consecutive nights (Figure S1b). The majority (626) of consecutive nights were probably spent within or near to the previous camp (*<*250 m apart). However, individuals moved more than 250 m on 87 occasions, corresponding to movements between camp locations on average every 8 or 9 days. The distributions of movements between consecutive nights for the two collections are shown independently in the insets in Figure S1d and e. Our GPS results support interview data in confirming a high mobility rate among the Punan Batu and regular movements between camp locations.

To examine the mobility patterns of the Punan Batu relative to other broadly defined hunter– gatherer populations, we compared our data with the Binford Hunter Gatherer dataset D-Place (2018a) annotated with Terrestrial Ecoregions of the World data D-Place (2018b) in the D-Place online dataset (Kirby et al., 2016). We extracted the *Distance moved per year (km)* (D-Place variable B014) and the *Number of moves per year* (B013) and divided these to estimate a metric of the average distance travelled when moving camp in order to make a comparison with our own data. We visualised the distribution of these variables with ecoregion for all ecoregions with *≥*10 society records (332 societies covering nine ecoregions). We note that societies follow various patterns of fishing, gathering and hunting, and that for some ecoregions societies will tend to be geographically clustered and phylogenetically related. Nevertheless, this comparative dataset represents a broad spectrum of human mobility lifeways covering a range of environmental contexts.

### Linguistic analysis

Swadesh word lists were video-recorded for two Punan Batu languages, Basa Latala and Punan Sajau. These word lists consists of a core vocabulary of 200 words (Table S2) with meanings that are assumed to be near-universal across human languages, and are commonly used in historical linguistics (Swadesh, 1955). The videotapes were transcribed into the International Phonetic Alphabet, digitised and combined with lists of Austronesian and Austroasiatic languages (Greenhill et al., 2008; Simons & Fennig, 2017; Lansing et al., 2007). Word lists were purposely chosen to be representative of these language families, and no attempt was made to provide exhaustive coverage because of the size and variation of the Austronesian language family in particular. The combined comparative dataset consisted of 37 word lists (Figure S7 and Lansing et al., 2020). Computational phylogenetic analysis involved calculation of pairwise Aline distances between words with shared meanings, the creation of a distance matrix assessing the average distance between each pair of languages and simple neighbour joining to infer tree topology. Consensus values for internal tree branches shown in Figure 5 were calculated by sampling with replacement from among glosses. These methods for computational linguistics analysis have been described previously (Downey et al., 2008, 2017). All linguistic datasets were manually curated and checked by a linguist. Manual examination of the Basa Latala and Sangiang word lists reveals no shared cognates, and little relationship with either Austronesian or Austroasiatic (Table S3). In all six cases where Sangiang has an Austronesian cognate, Ngaju Dayak does as well, which supports the hypothesis that any Austronesian vocabulary in Sangiang may be the result of contamination from Ngaju Dayak (or possibly from Malay via Ngaju Dayak).

In order to analyse the Latala language, the Swadesh list was used to elicit individual lexical items from two native Latala-speaking consultants. Indonesian was used as the prompt language and the sessions were video-recorded and later transcribed into the International Phonetic Alphabet. A total of 126 lexical items (63%) were collected. A second elicitation session was later conducted with a different set of language consultants in order to ensure the consistency of the elicited Latala lexical items. There is approximately 70% agreement between the two lists based on similarity between non-identical segments (see ‘Basa Latala’ in Lansing et al., 2020). Additionally, we conducted a bootstrap analysis of the complete data set including both Latala lists to examine the degree of agreement, with respect to the other language word lists included in the analysis (Figure S7). Because of the similarity of the Latala lists, we included only the lexical data from the first elicitation session in subsequent analyses. We explore morphological differences between these lists in the Supplemental Linguistics analysis.

## Supplementary diet information

Dietary information for 15 Punan Batu individuals (self-reported regularity of major consumed foods) was recorded during the biological sampling and medical clinic activity in October 2018 (Tables S4 and S5).

## Data Availability

The new genotype data have been submitted to the European Genome-Phenome Archive, hosted by the European Bioinformatics Institute (EBI) and the Centre for Genomic Regulation (CRG) (accession number EGAS00001004471). The complete Swadesh words list analysed in this work can be accessed on Figshare (doi: 10.6084/m9.figshare.12466001).

## Supplementary material

For supplementary material accompanying this paper visit https://doi.org/10.1017/ehs.2022.3. click here to view supplementary material
