# Supplementary material for: Deep ancestry of collapsing networks of nomadic hunter–gatherers in Borneo
Source: Evol Hum Sci. 2022 Feb 21;4:e9. doi: 10.1017/ehs.2022.3 (PMC10426063; doi:10.1017/ehs.2022.3)
Supplement: Supplementary file 1 [file S2513843X22000032sup001.docx]

Supplementary Materials for

Deep ancestry of collapsing networks of nomadic hunter-gatherers in Borneo

J. Stephen Lansing*∗*, Guy S. Jacobs*∗*, Sean S. Downey, Peter K. Norquest, Murray P. Cox, Steven L. Kuhn, John H. Miller, Herawati Sudoyo, John H. Miller,

Safarina G. Malik, Pradiptajati Kusuma*∗*†

*∗*These authors contributed equally to this work

†Corresponding author. Email: [pradiptajati.kusuma@gmail.com](mailto:pradiptajati.kusuma@gmail.com)

#### This PDF includes:

Supplementary Text Supplementary Figs. S1 to S7 Supplementary Tables S1 to S5

Linguistic data (Swadesh wordlists) are available at Figshare: (https://figshare.com/s/e6107567f0ef4a42bf27)

# Supplementary Text

## Supplementary Methods and Results on Linguistics

Note: The language data used in this analysis is available in Figshare: https://doi.org/10.6084/m9.figshare.12466001

When examining the two Basa Latala word lists, it is clear that the same form has been elicited, but with morphological differences. For example, the word for ‘to say’ was given as [luwai] in the first ses- sion, but as [paluwai] in the second – in this case, a verbal prefix [pa-] was included as part of the latter form (compare, for example, ‘to rub’ [parerit] and ‘to float’ [paliho] which also include this prefix). In other cases, there is variation in a single segment. The words for ‘child’, for example, were elicited as [majaN] and [bajaN], respectively. This is very likely the same form which is pronounced slightly differently by the two different consultants; in other cases, the difference may result from a transcription error, where certain phonemes are very similar to each other in terms of perception. A good example of this is the word for ‘to know’, which in the first session was recorded as [lakopan], and in the second as [parakopan] (also with the verbal prefix [pa-]), where the phonemes [l] and [r] are difficult to distinguish from each other perceptually. While there may be some question about the fact that there is only 70% agreement between the items elicited in the two sessions, this is actually not abnormal in this kind of situation where there may be more than one word which is used to express a particular concept. Slaska

[1] reports the following in a study in which English was used as the prompt language and Swadesh list data were collected from bilingual French and Polish speakers:

*In fact, out of the total 207 meanings, there were only 64 in French, and 48 in Polish, for which all the speakers agreed on the word which they provided [...] In both the French and the Polish studies, around a quarter of the meanings yielded 5 or more different lexemes: the exact figures are 46/207 (22%) for French, and 59/207 (29%) for Polish. Furthermore, it is noteworthy that there were 15 meanings for which fewer than half of the French sample (i.e. fewer than 20 speakers) agreed on any one particular lexeme; for Polish, the number of such meanings reached 21.* [1]

During our sessions, there were also a few cases where one lexical item was offered for more than one semantically-related prompt. For example, in the first session [pasa gata] is used for both ’all’ and ’you (pl)’; in another case, [pali kaban] was given for ’many’ in the first session, but was given for ’we’ in the second session. This kind of variation is normal for a natural language (and probably more so for a song language which is not used in everyday communication and is used in a more restricted context), and explains some of the discrepancies between the lists generated by our two elicitation sessions.

# Supplementary Figures

1. **B.**


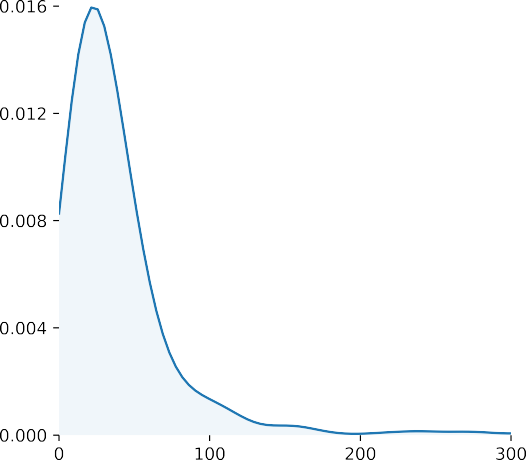


Movements < 250m Movements 250m

600

500

Total consecutive nights

400


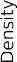
 300

200

100

2000 4000 6000 8000 10000 12000 14000

Distance travelled (m)


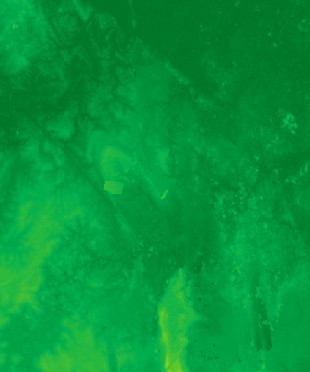

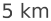

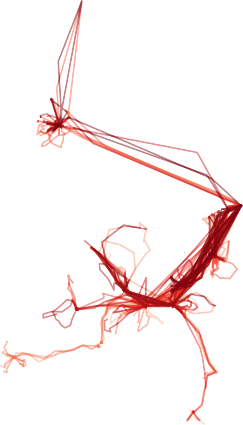


**D.**

300

15

0

0

0.2 0 5 10

Distance (km)

0

Count


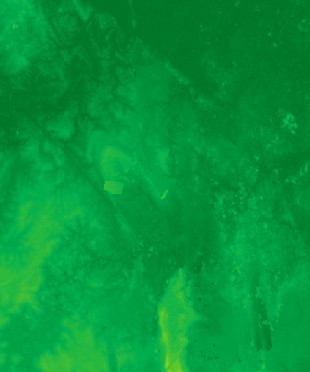

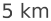

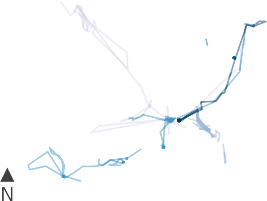


**C.**


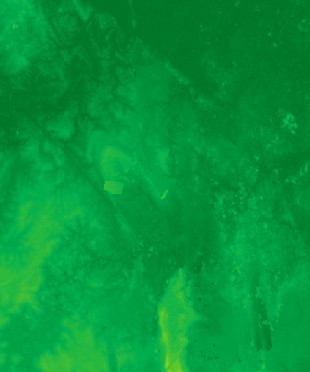

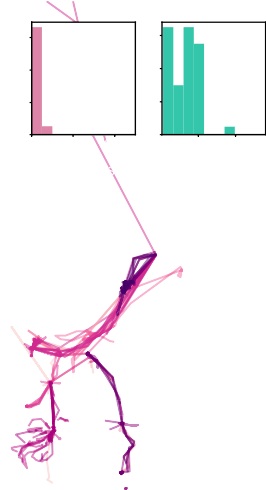

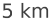


**E.**

**Fig. S1. Mobility analysis based on GPS readings. A)** Distribution of GPS location errors, based on the difference between simultaneous (*<*1min time difference) GPS readings from two devices carried by the same individual. **B)** Distribution of distances between consecutive night-time GPS locations, based on 713 consecutive nights from the 25 individuals included in the March and/or June 2019 data collections. GPS tracks recorded over three four-week periods, from **C)** October 2018 (5 individuals),

**D)** March 2019 (15 individuals) and **E)** June 2019 (17 individuals). Long straight lines are journeys by motorised canoe, or, for one data point leading to the north in June 2019, by road. A total of 27 individuals were included in this analysis, contributing 37 tracks. The distribution of distances between camp locations on consecutive nights is shown as an inset in **(D)** and **(E)** (left, moves *<*250m; right,

moves *≥*250m).


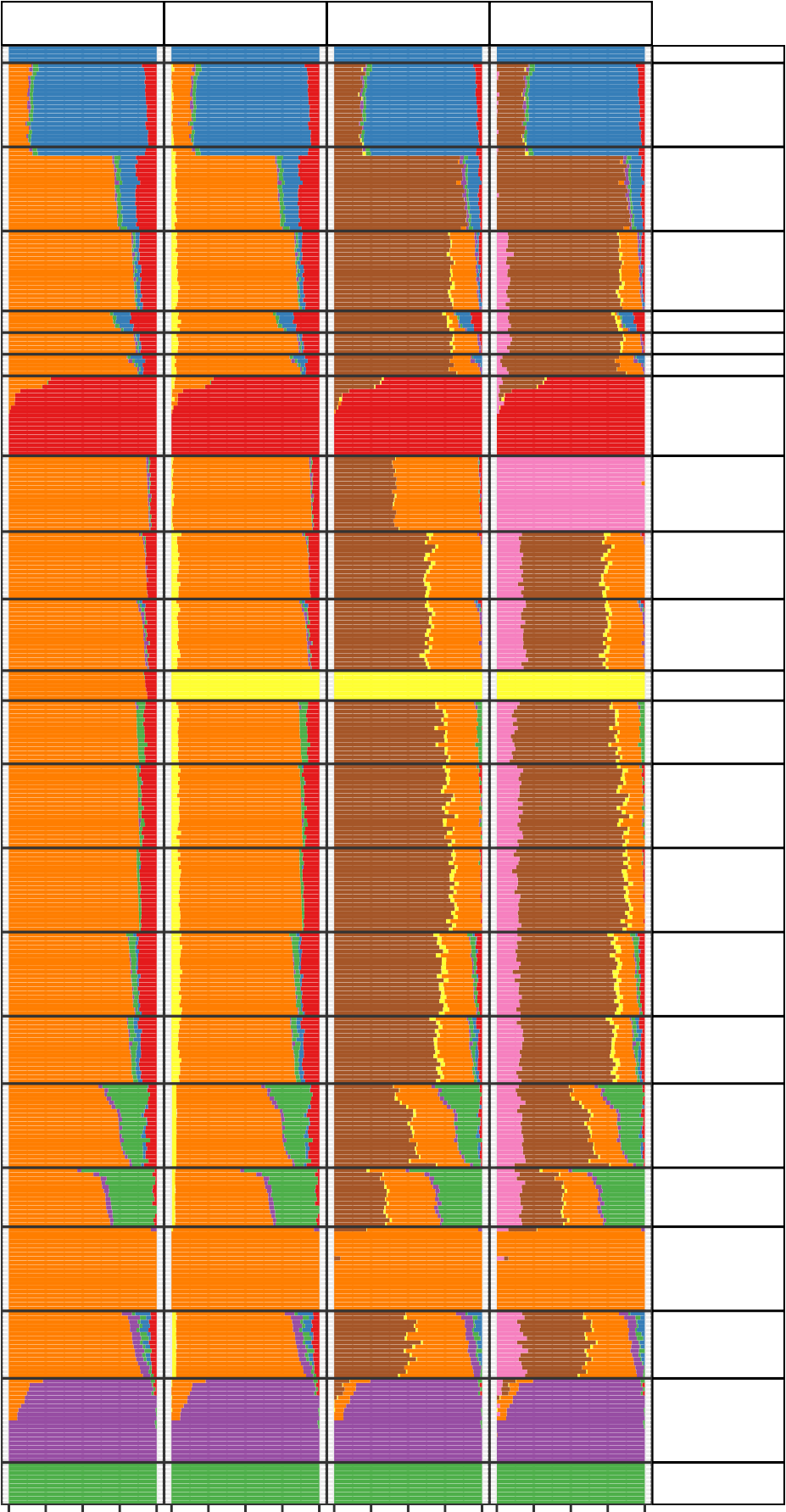


5

6

7

8

Mbuti

Brahmin

EastIndian

Vietnamese

Cambodian Dai Han

Jehai

Mentawai

Murut

Dusun

PunanBatu Lebbo

Samihim

Maanyan

Ngaju

Banjar

BajauKDR

NMaluku

Kankanaey

Filipino

Aeta

PNGHigh

**Fig. S2. Admixture plot from K=5 to K=8**. The "Punan Batu component" in the admixture plot first appears at K=7.

0.6

Murut

2.48

2.13

3.04

2.46

Dusun

5.59

3.68

**PunanBatu**

2.88

2.41

Lebbo

0.4

0.2

0.0

Density

0.6

Samihim

2.56

2.15

Maanyan

2.88

2.35

Ngaju

2.16

1.85

Banjar

2

1.73

0.4

0.2

0.0

0.0

2.5

5.0

7.5

10.00.0

2.5

5.0

7.5

10.00.0

2.5

5.0

7.5

10.00.0

2.5

5.0

7.5

10.0

IBD length (cM)

**Fig. S3. Individual IBD chunk length distributions of Borneo populations**. The mean (blue) and median (red) chunk length indicated.

800


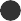

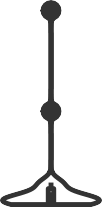

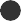

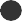

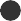

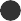


600

400

MB

200

0

**Fig. S4. Total runs of homozygosity (ROH) in the Punan Batu and comparison populations**. ROH is highest among the Punan Batu, but also elevated for other traditional hunter-gatherers (Jehai, Aeta) relative to agriculturalist groups.

0.100


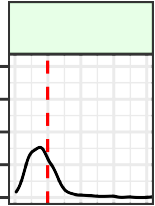


Murut

24.78


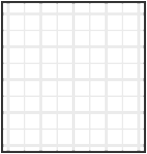


Dusun


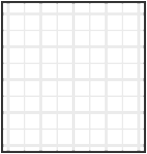


PunanBatu


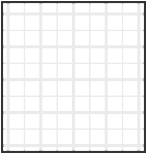


Lebbo


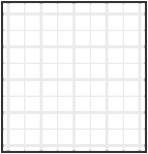


Samihim


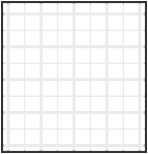


Maanyan


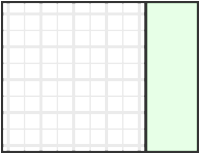


Ngaju

0.075

Dusun

0.050

0.025

0.000


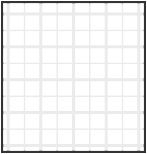

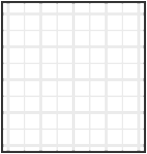

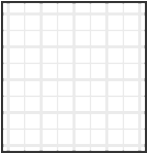

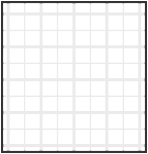

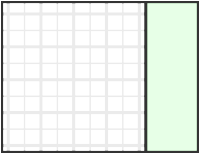
0.100


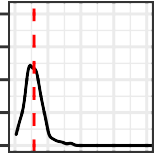


14.46


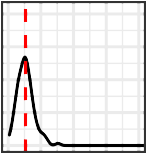


13.3

1

PunanBatu

0.075

0.050

0.025

0.000


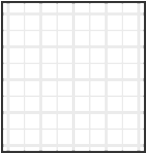

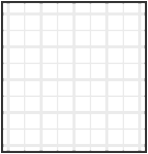

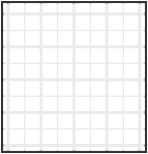

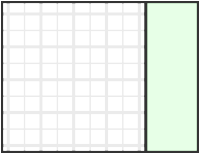
0.100


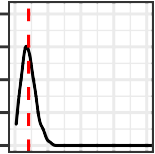


10.37


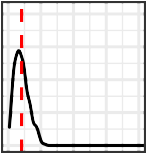


10.3

1


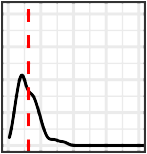


15.5

5

0.075

Lebbo

0.050

0.025

0.000


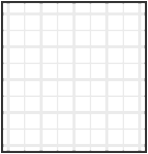

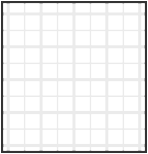

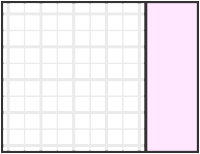
0.100


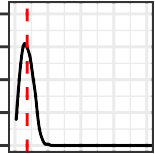


9.23


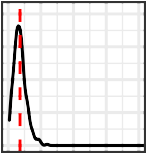


7

9.0


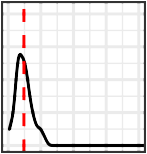


12.0

3


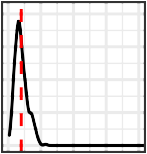


9

9.9

Samihim

density

0.075

0.050

0.025

0.000


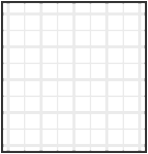

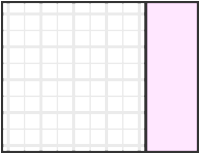
0.100


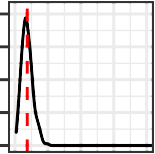


9.27


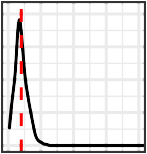


10


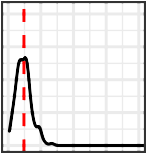


12.0

5


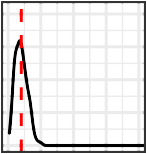


10.0

1


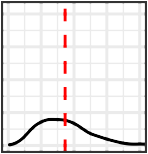


43.2

2

Maanyan

0.075

0.050

0.025

0.000


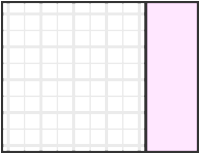
0.100


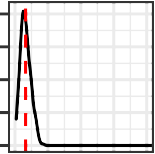


8.1


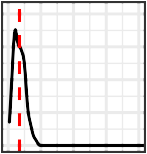


6

8.6


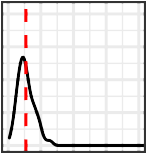


13.6

1


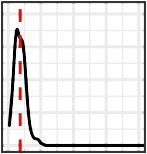


8

9.1


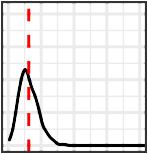


15.6

3


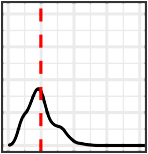


24.8

1

0.075

Ngaju

0.050

0.025

0.000

0.100


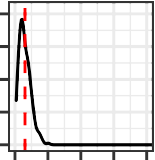


7.6


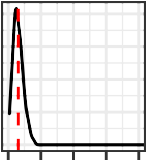


2

7.7


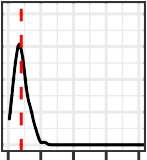


9

9.9


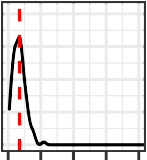


2

8.6


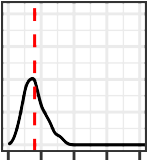


20.1

1


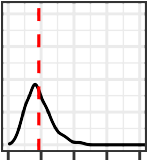


23.2

3


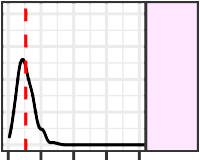


13.45

0.075

Banjar

0.050

0.025

0.000

0 2550751000 2550751000 2550751000 2550751000 2550751000 2550751000 255075100

### Total inter−pop pairwise IBD length (cM)

**Fig. S5. Identity By Descent (IBD) genetic sharing between individuals from different population pairs.** The Punan Batu show limited IBD sharing with other populations from Borneo. (Green: Popula- tions located in Northeast Borneo; Magenta: Populations located in South/Southeast Borneo)

#### A


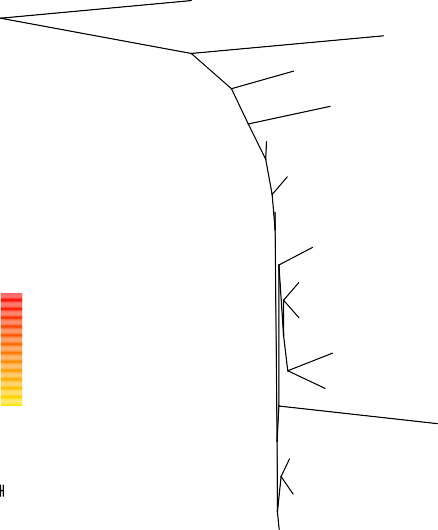


MbutiPygmy

PNGHigh

Aeta

Jehai

Cambodian Han

Banjar

Lebbo

Migration

weight

0.5

Murut

Dusun

Kankanaey Mentawai

0

Samihim

Maanyan

10 s.e.

Ngaju


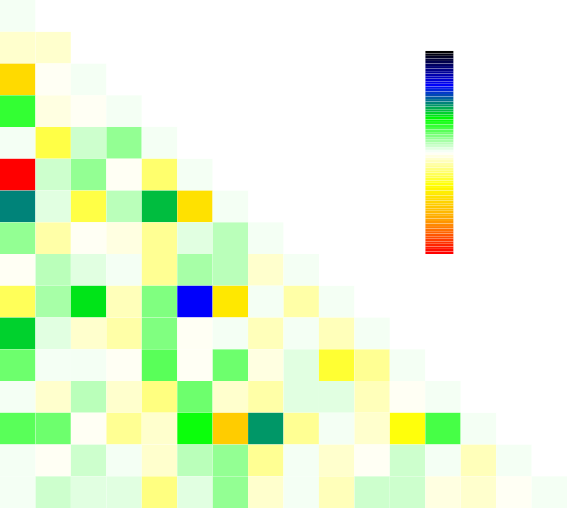


49.6 SE

−49.6 SE

Aeta

Banjar Cambodian

Dusun

Han Jehai

Kankanaey

Lebbo Maanyan

MbutiPygmy

Mentawai

Murut

Ngaju

PunanBatuSajau PNGHigh


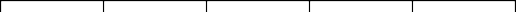


0.00 0.02 0.04 0.06 0.08 0.10

PunanBatuSajau

Samihim

Aeta

Banjar

Cambodian

Dusun

Han

Jehai

Lebbo

Maanyan

MbutiPygmy

Mentawai

Murut

Ngaju

PNGHigh

Samihim

Drift parameter

Kankanaey

PunanBatuSajau

#### B

Aeta


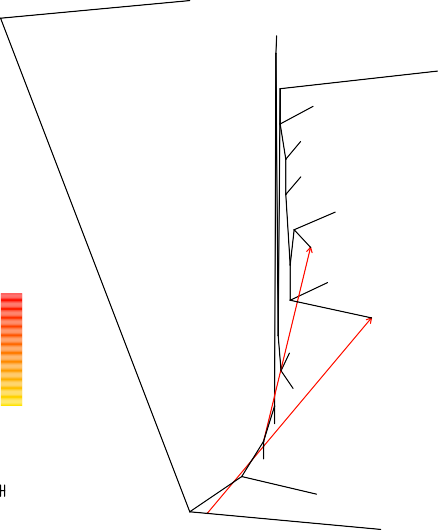


MbutiPygmy

Ngaju

Lebbo

Murut Dusun

Kankanaey

Han

Migration

weight

0.5

Mentawai

Aeta

Samihim Maanyan

0

Banjar

Cambodian

Jehai

10 s.e.

PNGHigh


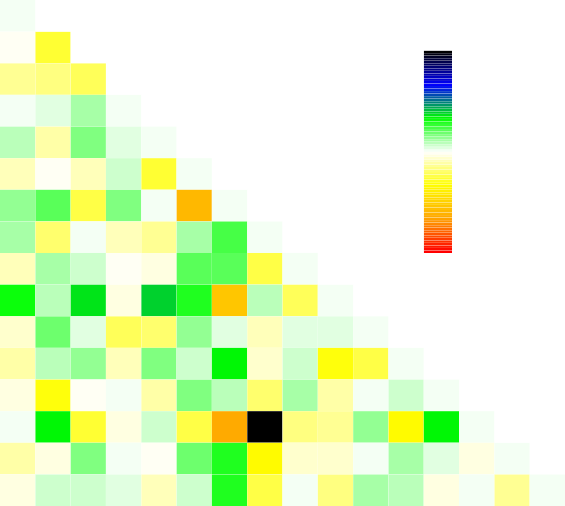


18.1 SE

−18.1 SE

PunanBatuSajau

Banjar Cambodian

Dusun

Han Jehai

Kankanaey

Lebbo Maanyan

MbutiPygmy

Mentawai Murut

Ngaju

PNGHigh

PunanBatuSajau

Samihim

Aeta

Banjar

Cambodian

Dusun

Han

Jehai

Kankanaey

Lebbo

Maanyan

MbutiPygmy

Mentawai

Murut

Ngaju

PNGHigh

PunanBatuSajau

Samihim


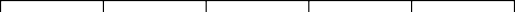


0.00 0.02 0.04 0.06 0.08 0.10

Drift parameter

####
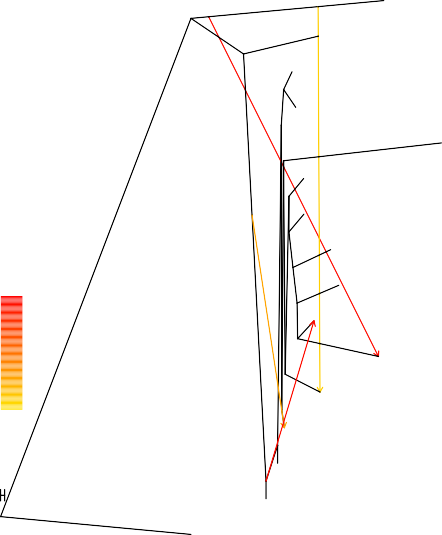
C

Jehai Samihim

PNGHigh

Aeta Banjar Cambodian


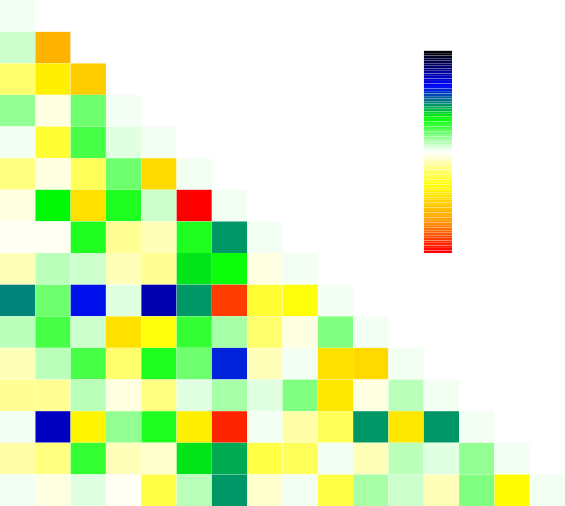


10.1 SE

−10.1 SE

Maanyan Dusun

PunanBatuSajau

Han

Migration weight

0.5

Murut Dusun

Mentawai Kankanaey

Han

Jehai Kankanaey Lebbo Maanyan MbutiPygmy Mentawai

Aeta Murut

0

Ngaju

Lebbo

Ngaju PNGHigh

10 s.e.

MbutiPygmy

Banjar Cambodian

PunanBatuSajau

Samihim

Aeta

Banjar

Dusun

Han

Jehai

Lebbo

Murut

Ngaju


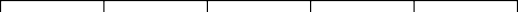


0.00 0.02 0.04 0.06 0.08 0.10

Cambodian

Kankanaey

Maanyan

MbutiPygmy

Mentawai

PNGHigh

PunanBatuSajau

Samihim

Drift parameter

#### D


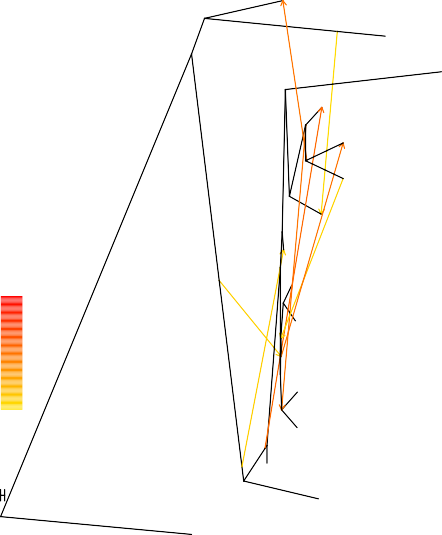


Aeta

PNGHigh

Han

Mentawai Kankanaey

Lebbo

Ngaju

Migration

weight

Samihim

1

Maanyan

Banjar

Dusun

0

Murut

Cambodian

Jehai

10 s.e.

MbutiPygmy


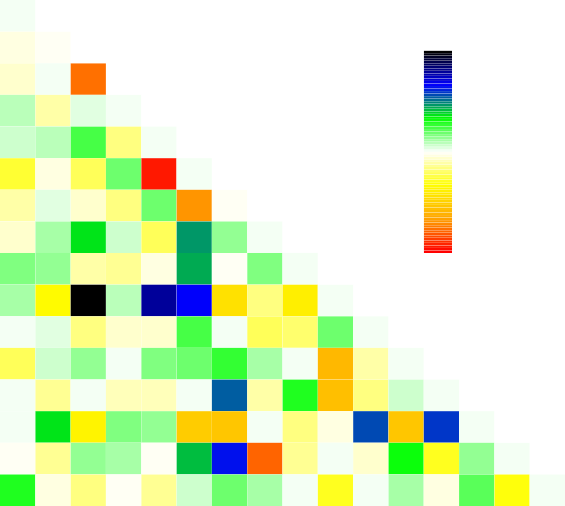


7.6 SE

−7.6 SE

Aeta

PunanBatuSajau

Banjar Cambodian

Dusun

Han Jehai

Kankanaey

Lebbo Maanyan

MbutiPygmy

Mentawai Murut

Ngaju

PNGHigh PunanBatuSajau

Samihim

Aeta

Banjar

Dusun

Han

Jehai

Lebbo

Murut

Ngaju


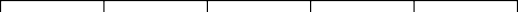


0.00 0.02 0.04 0.06 0.08 0.10

Drift parameter

Cambodian

Kankanaey

Maanyan

MbutiPygmy

Mentawai

PNGHigh

PunanBatuSajau

Samihim

**Fig. S6. Treemix analyses involving Punan Batu and other populations in ISEA and MSEA.** The Punan Batu appears to outgroup the Kankanaey-Mentawai cluster, and do not receive admixture nodes from MSEA-related ancestry.

Axis 2

−0.3−0.2−0.1

0.0

0.1

1. **Multidimensional scaling analysis 95% Confidence Ellipses**


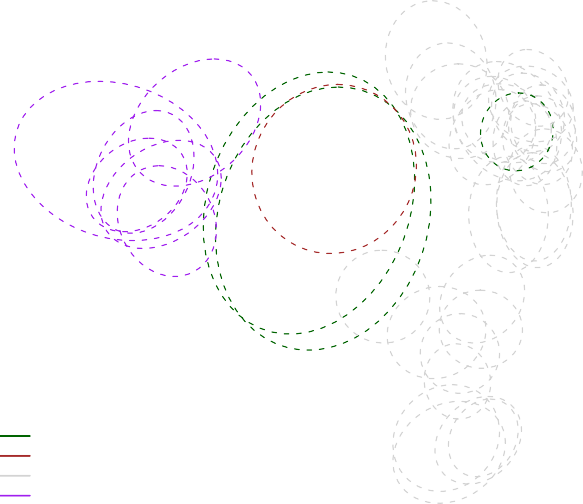


●

●

●

●

●

●

●

- ●Pu●nan Sajau

● **●**

●

● ●

●

●

●

- Sangiang

●

●

- Latala #2
  - Latala #1

●

● ●

●

●

●

●

●

●

●

Latala

Sangiang Austronesian Austroasiatic

● **●**

●


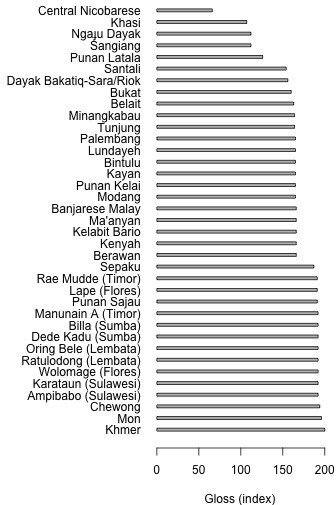


**A.**

0.2

0.3

−0.4−0.3−0.2−0.1 0.0 0.1 0.2

Axis 1

**Fig. S7. Additional linguistic analyses. A)** Data presence plot showing the total number of glosses for each language included in the linguistic analysis. **B)** Bootstrap analysis of Swadesh word lists. Language word lists are re-sampled by gloss with replacement and mean pairwise distances between languages are calculated (n=10,000). Classical multidimensional scaling and Procrustes rotation is used to estimate the primary axes of variation.

# Supplementary Tables

**Table S1. List of populations**

| **Population** | **Region** | **No of Inds** | **Source** |
| --- | --- | --- | --- |
| Mbuti | Africa | 4 | Li et al. 2008 |
| Brahmin | India | 20 | Mörseburg et al. 2016 |
| East Indian | India | 20 | Tätte et al. 2019 |
| Japanese | East Asia | 5 | Li et al. 2008 |
| Han | East Asia | 5 | Li et al. 2008 |
| Vietnamese | Mainland Southeast Asia | 19 | Mörseburg et al. 2016 |
| Cambodian | Mainland Southeast Asia | 5 | Li et al. 2008 |
| Dai | Mainland Southeast Asia | 5 | Li et al. 2008 |
| Jehai | Mainland Southeast Asia | 19 | Aghakhanian et al. 2015 |
| Mentawai | West Island Southeast Asia | 18 | New Data |
| Murut | West Island Southeast Asia (Borneo) | 16 | Mörseburg et al. 2016 |
| Dusun | West Island Southeast Asia (Borneo) | 17 | Mörseburg et al. 2016 |
| Punan Batu | West Island Southeast Asia (Borneo) | 11 | New Data |
| Lebbo | West Island Southeast Asia (Borneo) | 15 | Mörseburg et al. 2016 |
| Maanyan | West Island Southeast Asia (Borneo) | 20 | Kusuma et al. 2016 |
| Samihim | West Island Southeast Asia (Borneo) | 20 | Kusuma et al. 2017 |
| Ngaju | West Island Southeast Asia (Borneo) | 20 | Kusuma et al. 2016 |
| Banjar | West Island Southeast Asia (Borneo) | 16 | Kusuma et al. 2016 |
| BajauKDR | East Island Southeast Asia | 20 | Mörseburg et al. 2016 |
| Bugis | East Island Southeast Asia | 20 | Kusuma et al. 2017 |
| Mandar | East Island Southeast Asia | 20 | Kusuma et al. 2017 |
| N. Maluku | East Island Southeast Asia | 14 | Kusuma et al. 2017 |
| Sumba | East Island Southeast Asia | 20 | Cox et al. 2016 |
| Kankanaey | Philippines | 20 | Mörseburg et al. 2016 |
| Filipino | Philippines | 16 | Mörseburg et al. 2016 |
| Aeta | Philippines | 20 | Migliano et al. 2013 |
| Batak | Philippines | 20 | Migliano et al. 2013 |
| Mappi | New Guinea | 5 | New Data |
| PNG Highland | New Guinea | 10 | Li et al. 2008 |

**Table S2. Swadesh glosses used in linguistic analysis. (n=200)**

| I | thin | tail | to know | to tie | mountain |
| --- | --- | --- | --- | --- | --- |
| you (singular) | woman | feather | to think | to sew | red |
| he | man (adult male) | hair | to smell | to count | green |
| we | man (human being) | head | to fear | to say | yellow |
| you (plural) | child | ear | to sleep | to sing | white |
| they | wife | eye | to live | to play | black |
| this | husband | nose | to die | to float | night |
| that | mother | mouth | to kill | to flow | day |
| here | father | tooth | to fight | to freeze | year |
| there | animal | tongue (organ) | to hunt | to swell | warm |
| who | fish | fingernail | to hit | sun | cold |
| what | bird | foot | to cut | moon | full |
| where | dog | leg | to split | star | new |
| when | louse | knee | to stab | water | old |
| how | snake | hand | to scratch | rain | good |
| not | worm | wing | to dig | river | bad |
| all | tree | belly | to swim | lake | rotten |
| many | forest | guts | to fly | sea | dirty |
| some | stick | neck | to walk | salt | straight |
| few | fruit | back | to come | stone | round |
| ot her | seed | breast | to lie (as in a bed) | sand | sharp (as a knife) |
| one | leaf | heart | to sit | dust | dull (as a knife) |
| two | root | liver | to stand | earth | smooth |
| three | bark (of a tree) | to drink | to turn (intransitive) | cloud | wet |
| four | flower | to eat | to fall | fog | dry |
| five | grass | to bite | to give | sky | correct |
| big | rope | to suck | to hold | wind | near |
| long | skin | to spit | to squeeze | snow | far |
| wide | meat | to vomit | to rub | ice | right |
| thick | blood | to blow | to wash | smoke | left |
| heavy | bone | to breathe | to wipe | fire |  |
| small | fat (noun) | to laugh | to pull | ash |  |
| short | egg | to see | to push | to burn |  |
| narrow | horn | to hear | to throw | road |  |

**Table S3. Manual cognate count.**

|  | Austronesian | Austroasiatic | Sangiang | Basa Latala |
| --- | --- | --- | --- | --- |
| Austronesian | - |  |  |  |
| Austroasiatic | 0 | - |  |  |
| Sangiang | 6 | 0 | - |  |
| Basa Latala | 3 | 0 | 0 | - |

## Supplementary Diet Information

Dietary information for 15 Punan Batu individuals (self-reported regularity of major consumed foods) was recorded by staff of the Eijkman Institute for Molecular Biology during a medical clinic at the Datuk’s trading post on the Sajau River in October 2018.

| **Frequency** | **Rice traded** | **Rice grown** | **Wild tubers** | **Wild meat** |
| --- | --- | --- | --- | --- |
| Daily | 0.93 | 0 | 0.53 | 0.8 |
| Weekly | 0.07 | 0 | 0.33 | 0.13 |
| Infrequently | 0 | 0 | 0.13 | 0 |
| Unknown frequency | 0 | 0 | 0 | 0.07 |

**Table S 4:** Punan Batu self-reported regularity of major consumed foods. Daily indicates that a food is consumed at least once a day; weekly indicates that a food is consumed less than once a day but more than once a week; infrequently indicates that a food is consumed less than once a week; consumed indicates that the frequency of consumption was not reported by the interviewee.

| **Frequency** | **Rice traded** | **Rice grown** | **Wild tubers** | **Wild meat** |
| --- | --- | --- | --- | --- |
| Punan Batu | 0.93 | 0 | 0.53 | 0.8 |
| Punan Tubu (Close) | 0.07 | 0.98 | 0 | 0.63 |
| Punan Tubu (Remote) | 0 | 1 | 0 | 0.49 |

**Table S 5:** Daily consumption of different foods among the Punan Batu and Punan Tubu communities living near town and remotely. We compare information on rice sources and wild tuber sources with the broader category ‘starchy goods’ reported in Reyes-Garcia et al 2019, which includes both of these foods. Frequency of consumption at least once a day reported in each case; Punan Tubu totals calculated based on frequency * source (Tables 3 and 4 of Reyes-Garcia et al 2019)[2].

# References

1. N. Slaska, *Transactions of the Philological Society* **103**, 221 (2005).
2. V. Reyes-Garcia, *et al.*, *Food Security* **11**, 109 (2019).
